# Fixing Functional GI Disorders Using Microbes: Easier Said Than Done

**DOI:** 10.3389/fendo.2022.804179

**Published:** 2022-03-11

**Authors:** Gregor Reid, Raja Dhir, Peter A. Bron

**Affiliations:** ^1^ Centre for Human Microbiome and Probiotic Research, Lawson Health Research Institute, London, ON, Canada; ^2^ Department of Microbiology and Immunology, Western University, London, ON, Canada; ^3^ Department of Surgery, Western University, London, ON, Canada; ^4^ Seed, Venice, CA, United States

**Keywords:** gut-brain axis, probiotics, functional gastrointestinal disorders, microbial metabolites, *Lactobacillus* and *Bifidobacterium*

## Introduction

When we use the term ‘disorder’ to describe illness, it begs the questions what is ‘order’ and what happened to it? In attempting an answer, we have to consider the features of the disorder. Functional gastrointestinal disorders (FGIDs) are complex, multi-system phenotypes with an array of presentations and comorbidities (1). These include dysmotility, visceral hyperalgesia, autonomic nervous system dysfunctions, familiarity, psychosocial triggers, and postinfectious events. The inference is that the orderly state is not terribly resilient or stable. Yet, such a conclusion would be doing a disservice to evolution given that most humans do not suffer from gastrointestinal disorders.

To understand these conditions, investigations have focused on physiological and psychological factors or the so-called bidirectional communication pathways that render the term “gut-brain axis” (2). This axis initially only referred to the connection between the nervous system and gut function, but it has been expanded to include native gut microbiota. This concept was supported by rodent studies showing a role for the vagus nerve in transferring microbial signals that influence brain responses (3, 4). This has stemmed from appreciating the preponderance of microbes in the gut which in turn led to hypothesizing that microbial metabolites hold the key to resilience or disruption.

This is essentially a reductionist approach that ignores the other microbes in the body, including ones linked through the vagus nerve (5). It suggests that the microbial products have a significant influence on the brain, but do we have sufficient data to prove that? An excellent recent review suggests not in humans (6; see Table 2 showing only mouse data). To date, there have been no measures published on neurotransmitter levels in the blood and brain of humans post-fecal microbiota transplant (FMT) or probiotic treatment. Some tools exist as demonstrated by the use of diffusion magnetic resonance imaging (MRI), functional magnetic resonance imaging (fMRI), and positron electron tomography to measure functional effects of serotoninergic receptor (5-HT_2A_R) stimulation by a naturally occurring psychedelic prodrug, psilocybin (7). A number of microbes have been hypothesized as potentially being able to affect human brain chemistry from the gut and related FGIDs. Strains of bacteria potentially useable as probiotics are known to produce neurochemicals including gamma-aminobutyric acid (GABA), serotonin and acetylcholine (8). One recent study has shown the existence of GABA-producing pathways in *Bacteroides*, *Parabacteroides* and *Escherichia* species present in the gut (9) which makes it more difficult to prove that the ingestion of a probiotic lactobacilli was directly responsible for higher levels of this inhibitory neurotransmitter. Interestingly, this paper reported use of fMRI to show that lower levels of GABA-mediated responses in patients with major depressive disorder correlated with low abundance of fecal *Bacteroides*. This species has been proposed as a probiotic, but given its pathogenic potential no *B. fragilis* strain has yet been brought to market after rigorous human testing.

It should be noted that patients with functional disorders such as irritable bowel syndrome (IBS), have visceral and cutaneous hyperalgesia (10). This spinal innervation of the gut is important as are endocrine and immune signals in terms of the gut-brain axis. However, the potential for microbiome-targeted interventions has not been well explored in humans. One small study using one particular probiotic strain showed no effect on viscerperception in IBS patients (11) but whether this is true for other probiotic strains remains to be seen. A possible exception could be *Limosilactobacillus* (formerly *Lactobacillus*) *reuteri* DSM 17938 which can relieve colic when given with breast feeding (12) and it has shown significant reduction in functional abdominal pain intensity in children, though the mechanisms are unknown (13).

Overall, the link between microbes and the brain and functional gut disorders has led to interest in applying probiotics, FMT, prebiotics, fermented foods or synbiotics to re-establish intestinal homeostasis. However, much more experimental effort must be exerted to justify the cost of extended clinical trials. It is our view that three aspects require attention before embarking on clinical studies: the timing of application and associated evidence therein; the selection and design of the intervention; and the experimental approaches deployed.

## The Life Spectrum and Timing

It has been well documented that humans inherit microbes from birth and that many of these early colonizers remain in place through life and are difficult to displace (14). There are certainly studies showing disruption to the gut microbiota of infants, particularly with prematurity (15) certain antibiotic exposure (16) affecting education of the immune system with consequences later in life (17). Although difficult to prove, there is growing evidence that FGIDs in adulthood have origins in infancy (18, 19). Thus, for FGIDs that are microbial-related and the markers for disorder identified in early life, efforts could be made in infancy to prevent the disruptive organisms from taking hold. One option could be to implant beneficial microbes at a young age. In addition to all the ethical issues, a prerequisite for such an intervention would be proving that the instability that occurs later in life was due to an aberrant microbial colonization during microbiome development.

One way to investigate this would be to examine medical data of FGID patients and compare those who had been given probiotic-supplemented formula, human milk or antibiotics in infancy. Secondly, medical records could be examined to attempt to determine when the triggers occurred that disrupt the equilibrium sufficiently to cause one or more of the undesired FGID manifestations. Such utilization of patient records can be very informative (20).

Genetic elements are believed to be playing a role in the enteric nervous system (21), and one study using patient questionnaires that included participants not diagnosed with IBS, proposed a link between six genetic susceptibility loci and IBS (22). Nevertheless, despite the promise of human genetics the causes of FGID do not appear to be primarily genetically linked. That places importance on the immune response and the microbiome, particularly metabolites and the strains producing them.

Attempting to correct a disorder rooted in events of childhood is an onerous task. Freeze dried probiotic organisms can be useful to confer benefits in patients with dysmotility (23) without being able to colonize the gut unless it was evacuated as happens when FMT is being administered.

Some studies have shown that the older we get the more the microbiome deteriorates through losing species and resilience (24). However, one study of the gut microbiota also found similarities between centenarians and young adults (25). This then raises questions of whose feces would be appropriate for implantation in FGID patients? From a safety and “ecological restoration potential” perspective this would ideally be the autologous microbiome derived from fecal samples collected from a symbiotic state, prior to the occurrence of the FGID. To this end, acute myeloid leukemia patients treated with chemotherapy combined with a broad-spectrum antibiotic regime typically suffer from gut microbiota dysbiosis but displayed restored microbial communities in a one-armed phase II clinical trial after autologous FMT (26). Obviously FGID cannot be predicted to occur in the near future in most situations other than scheduled antibiotic treatment. This complicates the timing of collection of symbiotic autologous FMTs, especially when considering the fact that long term storage prior to re-inoculation might be detrimental to specific microbiota members, leaving no other option than the employment of allochthonous FMTs.

## The Intervention

The methods used to identify donors for FMT are extensive and costly (27), such that most centers have a small core of providers. The stool is collected because the donor is healthy not because his or her microbes are suitable to counter certain disorders. This could be interpreted that only the metabolites need to be transplanted, but the types and amounts necessary to confer benefits are unknown and the active metabolism of the transplanted organisms appears to be vital. The primary outcome is the restoration of health in the recipient, but the mechanisms are far from understood. The SHIME and other three-stage continuous culture systems could be used to examine the dynamic function of the fecal microbiota (28, 29) and provide a rationale for using different feces for different conditions and supplementing with specific probiotic strains.

As studies on probiotic strains are indicating, organisms of the same species do not necessarily have the same functional characteristics (30, 31). To address this, donor stool could be examined to identify an appropriate one for the FGID condition, or probiotic strains such as *Lactiplantibacillus* (*formerly Lactobacillus*) *plantarum* LP01, *Bifidobacterium breve* BR03, *Bifidobacterium animalis* subspecies *lactis* BS01 and *Bifidobacterium infantis* 35624 shown to alleviate dysmotility (23, 32), could be spiked into the product prior to instillation. Unfortunately, the latter would create regulatory issues and require safety studies despite FMT and these probiotic strains being permitted for human use separately.

The ability to identify microbiome changes that are associated with disease or a return to health would be challenging and certainly require multiple sampling over time, use of various ‘omics’ techniques, and assessment of confounding factors including diet, medications and life events that may induce stress. Fecal sampling is not ideal but until a device can collect material from the gut in real time without the use of laxatives, and until methods can identify microbiome structure and co-dependencies (33), gaps will remain in understanding resilience and early signs of disorder.

It has been proposed that the instilled microbes should target specific neuroendocrine signaling pathways (34), but there is a severe paucity of data from humans indicating which specific pathways can be targeted by which microbes or by the molecules they produce. This is all the more complex given that they include autonomic, neuroendocrine, enteric, and immune systems as well as the hypothalamic-pituitary-adrenal axis (35). Other host factors such as gender and body mass index, not adequately considered to date, could influence the composition of the gut microbiota (36).

Whichever intervention is to be tested, it is critical that strain characteristics are known and align with the desired end point. Thus, if the subject has a deficiency in serotonin, the organism or fecal material used should be shown to produce it *in vitro*. If this release of molecules such as serotonin is how gut microbes influence the brain, it raises the question of whether flooding the circulation with neurotransmitters would be effective. Indeed, it could be detrimental to the workings of the nervous system which utilizes spatially restricted transmission processes. Furthermore, it has long been known that neurotransmitters and amino acid precursors can be accumulated by human blood platelets and thus less likely to make a major impact on the brain (37).

It is our belief that too many studies have been performed on rodents with an implied correlation to humans and concluding that the gut-brain axis is hugely influential (38). While there are certainly some experiments that cannot ethically be performed in humans, much of what we have discussed can and should be done in humans through observational, cohort and/or blinded randomized trials (5). The use of probiotics, prebiotics, synbiotics and FMT is already widespread and with any study requiring ethics board approval, and in some cases regulatory approval, careful inclusion and exclusion criteria should ensure appropriate subjects are enrolled. As with any intervention, risk/benefit must be assessed, and safety monitored during the trial (39).

One recent meta-analysis revealed no significant difference in the symptoms of schizophrenia, stress, and anxiety between probiotic and placebo groups, post-intervention, with only an effect on patients with depression (40). This further emphasizes the need to perform not only large studies over longer periods of time, but ones that use strains selected with appropriate properties, samples taken to examine neurotransmitter levels before, during and after treatment, and to have a comparative arm with a serotonin uptake inhibitor. We encourage granting agencies to consider this when funding future studies in this area.

## The Use of Imaging and Real-Time Sampling

The use of imaging technologies aligned with spectroscopy and compound labeling might identify molecules released by gut microbes that reach the human brain and/or induce a favourable response (41–43). Such studies, or alternatives, will be expensive and time-consuming but necessary. This will require a team approach that assesses the confounders, cognitive function, behaviour, the microbes and metabolites with sampling done as close to the favourable response as possible. An ongoing study is looking into brain-gut-microbiota-axis and its effect on cognition as well as anatomical and functional brain connectivity in patients with IBS (44).

There are precedents with real-time sampling of the intestine upon short-term ingestion of probiotics and use of MRI post-consumption of yoghurt (45–48), though desired clinical endpoints will likely require much longer-term administration. Various techniques, such as positron-emission-tomography (PET) (49), functional magnetic resonance imaging (fMRI), voxel-based morphometry (VBM: to gray matter volume), diffusion tensor imaging (DTI: to visualize microstructural details of white matter), fractional anisotropy (FA: to assess directional coherence of water diffusion within tissues) (50) are being developed to examine brain function and structural connectivity, and could perhaps be useful following microbial ingestion. Hybrid PET-MRI also has potential by providing anatomical, functional, metabolic and biochemical data on the brain (51). However, an issue that will be difficult to resolve is proving that any effects that are seen can be proven to be due to gut microbes or indeed to ones from other sites, including the urogenital tract, lungs and nasopharynx. The use of labelled bacteria and developing imaging strategies to follow their progression through the intestinal tract is being studied (52), though the size of the organisms makes resolution challenging, and then determining that their presence is responsible for changes in brain function will again prove difficult. Nevertheless, these studies are worthy of pursuit as are those which can provide information in real time. Experiments with quantum dots as drug tracers (53) might prove useful if they could be applied to bacterial metabolites or molecules in the brain that are influenced by them.

## In Summary

There is growing interest in not only microbes playing a role in triggering functional gastrointestinal disorders, but also in use of beneficial ones to restore health. In approaching this topic scientifically and then translating this to clinical studies, it is important to understand what strains are doing in the gut and how their metabolites affect distant sites including the brain and central nervous system. New treatment options are becoming available and with the use of various imaging, microbiological and genetic tools and study sample sizes that are appropriate, it will be possible to identify which interventions have value and which do not. Given the broad nature of functional gut disorders, microbes should not be expected to be a magic bullet.

## Author Contributions

Manuscript drafted by GR, edited and revised by RD and PAB. All authors contributed to the article and approved the submitted version.

## Conflict of Interest

RD is co-owner of Seed and PAB is an employee. GR consults for Seed and KGK Science.

## Publisher’s Note

All claims expressed in this article are solely those of the authors and do not necessarily represent those of their affiliated organizations, or those of the publisher, the editors and the reviewers. Any product that may be evaluated in this article, or claim that may be made by its manufacturer, is not guaranteed or endorsed by the publisher.
